# Impact of a Telenursing service on satisfaction and health outcomes of children with inflammatory rheumatic diseases and their families: a crossover randomized trial study protocol

**DOI:** 10.1186/1471-2431-14-151

**Published:** 2014-06-18

**Authors:** Anne-Sylvie Ramelet, Béatrice Fonjallaz, Joachim Rapin, Christophe Gueniat, Michaël Hofer

**Affiliations:** 1 Institute of Higher Education and Nursing Research, University of Lausanne, CHUV, Rte de la Corniche 10, Lausanne 1011, Switzerland; 2 Haute Ecole de Santé Vaud (HESAV), University of Applied Sciences and Arts Western Switzerland, Rte de la Corniche 10, Lausanne 1011, Switzerland; 3 Geneva League for Rheumatology, La ligue Genevoise contre le Rhumatisme, Rue Merle d’Aubigné 22, Geneva 1207, Switzerland; 4 Département Médico-Chirurgical de Pédiatrie (DMCP), CHUV, Rue du Bugnon 21, Lausanne 1011, Switzerland

**Keywords:** Telenursing, Hotlines, Nursing, Child, Health outcomes, Rheumatic diseases

## Abstract

**Background:**

Pediatric rheumatic diseases have a significant impact on children’s quality of life and family functioning. Disease control and management of the symptoms are important to minimize disability and pain. Specialist clinical nurses play a key role in supporting medical teams, recognizing poor disease control and the need for treatment changes, providing a resource to patients on treatment options and access to additional support and advice, and identifying best practices to achieve optimal outcomes for patients and their families. This highlights the importance of investigating follow-up telenursing (TN) consultations with experienced, specialist clinical nurses in rheumatology to provide this support to children and their families.

**Methods/Design:**

This randomized crossover, experimental longitudinal study will compare the effects of standard care against a novel telenursing consultation on children’s and family outcomes. It will examine children below 16 years old, recently diagnosed with inflammatory rheumatic diseases, who attend the pediatric rheumatology outpatient clinic of a tertiary referral hospital in western Switzerland, and one of their parents. The telenursing consultation, at least once a month, by a qualified, experienced, specialist nurse in pediatric rheumatology will consist of providing affective support, health information, and aid to decision-making. Cox’s Interaction Model of Client Health Behavior serves as the theoretical framework for this study. The primary outcome measure is satisfaction and this will be assessed using mixed methods (quantitative and qualitative data). Secondary outcome measures include disease activity, quality of life, adherence to treatment, use of the telenursing service, and cost. We plan to enroll 56 children.

**Discussion:**

The telenursing consultation is designed to support parents and children/adolescents during the course of the disease with regular follow-up. This project is novel because it is based on a theoretical standardized intervention, yet it allows for individualized care. We expect this trial to confirm the importance of support by a clinical specialist nurse in improving outcomes for children and adolescents with inflammatory rheumatisms.

**Trial registration:**

ClinicalTrial.gov identifier: NCT01511341 (December 1^st^, 2012).

## Background

Pediatric rheumatism represents a large group of inflammatory and non-inflammatory diseases of the locomotion system. Most of the children affected by these diseases present a chronic course and this can have a significant impact on their quality of life. Rheumatic diseases can have long-term effects on patients’ lives and may interfere with their schooling, their later working life, as well as with family functioning. Over the last decade, the treatment of inflammatory rheumatic diseases has significantly improved thanks to the use of new biological agents, although their therapeutic benefits can be accompanied by significant undesirable side effects. These new drugs are also expensive and thus participate significantly to the burdens these diseases place on health spending and society. To offer high quality care to these children and their families, it is essential to promote the best possible quality of life and limit the financial and social costs of pediatric rheumatic diseases.

According to recent data from the Swiss registry of pediatric rheumatic diseases, the annual incidence of all patients seen by the nine national centers was 40.6 new patients per 100,000 children, with 56.8/100,000 in the Canton of Vaud (western Switzerland) [1]. Close to two thirds of these patients (n = 2,120) were diagnosed with an inflammatory disease. These can be classified into three groups: juvenile idiopathic arthritis (JIA), connective tissue diseases (CTD), and other inflammatory diseases [1]. JIA is the most common childhood inflammatory rheumatic disease and is an important cause of short- and long-term disability [2,3]. The typical clinical symptoms of JIA last for a minimum of 6 weeks and include persistent swelling of one or more joints, limited range of motion in the joints, pain during movement, and inflammation that may last for years until adulthood [4]. Fever, reduced physical activity, poor appetite, and flu-like symptoms are also clinical features of patients suffering from JIA [5]. JIA is a heterogeneous disease comprising seven different categories, and disease severity varies widely between patients [6]. Inflammation associated with JIA and other rheumatic diseases can result in significant chronic pain [7,8], decreased functional ability [9], impaired physical development [10], and decreased overall well-being and quality of life [11,12]. The disease and its associated treatment challenge children and their families daily; children face altered body image, anxiety from teasing and social nonacceptance, fears about the course of the disease, and uncertainty about their future [13]. The PRINTO study showed significant physical impairment and suboptimal psychosocial functioning in children with JIA when compared to healthy matched-controls. In the JIA children, physical status was mostly altered by the level of disability, whereas psychosocial health was more affected by chronic pain [14,15]. In addition to clinical symptoms, inflammatory rheumatic diseases, like any chronic disease, have a significant impact on the functioning of the family as a whole and create significant distress for parents [16]. As Hopia stated, “parental well-being is governed by the child’s illness. When a child has a chronic illness, the whole family is ill” (p.191) [17]. Families with chronically ill children not only have to learn how to adjust to their child’s needs, but also how to mobilize their resources to maintain their own health and positive mental images, and manage their uncertainty, anxiety, and distress [16,18].

In summary, the consequences of inflammatory rheumatic diseases on children and their families are significant. Up to 75% of these children go on to suffer from symptoms or complications related to the disease in adulthood [19-21]. Long-term follow-up of adults who had suffered from JIA showed they often had significant levels of disability over prolonged periods related to the ongoing active disease [22], as well as social dysfunction compared to the general population [23]. This was manifested in higher rates of unemployment, shorter timespans lived with a partner, and decreased fertility rates [23].

Caring for children with chronic rheumatic diseases involves a multidisciplinary approach [24]. As there is currently no cure for JIA, disease control and symptom management become of foremost importance to minimize disability and pain [11]. The key elements to achieving optimal outcomes consist of early symptom detection and diagnosis, disease stabilization, aiming for remission, and concordance between the different treatments and interventions [25]. Stabilization of the disease involves pharmacological treatment to control inflammation and pain, reduce disease progression, joint damage, disability and loss of function, and achieve remission. Pharmacological therapy relies on various combinations of non-steroidal anti-inflammatory drugs, analgesics, corticosteroids, disease-modifying anti-rheumatic drugs and biological response modifiers such as anti-tumor necrosis factor [26-28]. Special care should be applied to dealing with children, as susceptibility to the toxicity of these drugs can differ considerably between individuals and types of treatment [27]. Individualized monitoring and management of these drugs’ side effects is important as the type of drug therapy can have an impact on nutrition, development, internal organ damage, growth development and risk of infection.

Non-pharmacological approaches aim mostly at relieving pain, decreasing stiffness, and avoiding pain recurrence [26]. It involves physiotherapy to help prevent malalignment and improve function [25]. Other non-pharmacological treatments for pain have been reviewed by Kimura et al. [12]. They revealed that cognitive behavioral therapy, physical therapy and exercise, and other approaches (massage) were promising therapies for relieving pain and constituted an important part of the treatment as well as of the multidimensional approach to pain management. Other complementary or alternative treatments—such as hot/cold aids, transcutaneous electrical nerve stimulation, natural medicine, and massage—have been shown to improve comfort [25] and patients need for more information about them [29].

The recommended assessment of patient health in order to monitor progress of the disease includes not only an appraisal of physical features, but also a health-related quality-of-life measurement [30,31]. Overall assessment of JIA’s disease activity consists of a core set of measurements, including physician’s and parent/adolescent’s overall assessments of disease activity, a count of joints with arthritis and limited movement, a functional assessment, and the erythrocyte sedimentation rate [30]. The equivalent disease-specific assessments are available for chronic inflammatory rheumatic diseases other than JIA [31]. Due to the potential rapid change in disease activity, this health assessment is usually performed every three to six months [32].

In addition to the medical visits, newly-diagnosed patients with complex needs require close monitoring, thus follow-up is an important aspect of care for these patients and their families. They need time to adjust to the new diagnosis, the practicalities of the treatment, and to cope with fears and uncertainty for the future [33]. Follow-up monitoring of care, in which nurses play a key role, aims to anticipate, identify and prevent problems at a clinical level [34,35] or other psychological, emotional, and social problems related to the disease [36].

The supporting role of nurses in the care of these children aims to limit the potential for further disability and psychological complications [37,38]. They play particularly key roles in supporting the specialist teams caring for patients with rheumatic diseases, recognizing poor disease control and the need for changes in treatment, providing a resource for patients on treatment options and how to access additional support and advice, and identifying best practices with which to achieve optimal outcomes for patients and their families [25], p.48.

To fulfill their role, rheumatology nurses need to have specific knowledge and competencies in the physical and psychosocial evaluation of patients, the development and implementation of treatment plans, therapeutic education, and research utilization [34,39]. They should also have the interpersonal skills to be able to respond to both the child’s and the parents’ psychological and affective needs, and help the parents cope with their child’s illness [38]. The developmental stages that children go through during their disease is specific to pediatric rheumatology, resulting in the need to adapt nursing interventions to children’s ages. For instance, nurses have to develop strategies for adolescents to cope with chronic illness that fit into their adolescent lifestyles [40,41].

In their supporting role, nurses are also the link between medical practitioners, other healthcare professionals, and families; they therefore play a key role in the follow-up care. Follow-up with children and their families can be ensured by regular telephone consultations made by experienced, specialized rheumatology nurses. The following paragraph introduces telenursing (TN)—a nurse-led intervention adopted by many healthcare providers to increase their efficiency in meeting patients’ needs [42].

TN includes a wide range of activities including assessing patients’ needs, conducting triage in emergencies, reassuring callers, providing nursing advice, teaching, providing medical information, and referring patients to appropriate care at an appropriate location [43,44]. TN services aim to establish a relationship with the caller, identify the concern, assess the condition, solve problems in collaboration with the caller, and select appropriate solutions [44]. TN has been used in different ways, settings, and purposes. A comprehensive search of the literature revealed numerous articles related to TN: on triage [45-48]; state and national help lines [49-51]; follow-up of specific health conditions such as pregnancy [52,53]; post-operative care [54,55]; medication adherence [56,57]; and chronic illness [58-60]. Most studies concerned adults, but some also involved pediatrics. The following paragraph discusses TN with an emphasis on pediatric studies involving children with chronic disease.

Follow-up calls to patients with *chronic diseases*, such as asthma, heart conditions, diabetes, or cancer have been well evaluated in adult populations [58,60,61], including one systematic review [59]. In this latter review, nurse-led interventions, such as telephone consultations, showed some benefits on medication adherence by patients with type-2 diabetes. This type of follow-up service has not been commonly used in pediatric populations requiring long-term follow-up of their health conditions, and this despite the drastically increasing number of children with chronic diseases or needing long-term care after a life-threatening condition [62]. A literature search found only two pediatric studies that evaluated this type of telephone service. Gischler et al. evaluated the frequency and the nature of the calls made by parents of children born with severe anatomical congenital anomalies to a 24-hour telephone helpline [63]. A total of 670 calls occurred outside office hours: 24.5% calls by nurses, 20.2% led to a consultation with the emergency department, resulting in 4.9% admissions. A 24-hour helpline provides easy access to medical information and offers supportive care to parents at relatively low cost. This nurse-led telephone intervention proved to be safe and efficient when back-up by a pediatric physician was provided [63], p.625. Letourneau et al. described the use of a TN line in a pediatric neurology clinic [64]. Most of the calls concerned problems related to epilepsy and nurses were able to solve half of the problems without requiring further medical intervention. Although these two studies are descriptive in nature, and thus the results should be interpreted with caution, they demonstrate that a TN line may indeed assist in the provision of care and support to complicated subspecialty patients.

Ensuring follow-up for children with rheumatic diseases is critical for the physical and psychological wellbeing of both child and family. Studies in pediatric settings are scarce and to the best of our knowledge nonexistent in pediatric rheumatology. Finally, the effectiveness of such a service remains to be proven in children with chronic rheumatic diseases. In addition to the satisfaction outcome commonly used in previous studies, the inclusion of patient-oriented outcome measures, such as health status, would greatly add to the value of future research in this area.

### Study objectives and hypotheses

This study aims to evaluate the effect of a telenursing intervention on the satisfaction and health outcomes of children with inflammatory rheumatologic diseases and their families. The primary objective is to evaluate the effects of TN on the children/adolescent’s and parents’ satisfaction with the care given for inflammatory rheumatic diseases. The secondary objectives are to evaluate the effects of TN on the child’s clinical health status, quality of life, treatment adherence, and service utilization.

## Methods/Design

### Study design

This study has a randomized crossover, experimental longitudinal design, in which the intervention (telenursing) is evaluated with the same subjects and so eliminates between-subject variability, in particular due to the heterogeneity of the disease [6,65]. Crossover trials are particularly useful when the outcomes of interest are symptoms and functional capacity [66].

### Setting and participants

The setting is the pediatric rheumatology outpatient clinic of a tertiary referral hospital in the Canton of Vaud, which is part of the Pediatric Rheumatology Network of Western Switzerland. Approximately 110 new patients are admitted each year, of which about 50 present with chronic inflammatory rheumatic diseases.

The target study population will consist of children meeting the following criteria, plus one of their parents (or their legal guardian).

Inclusion criteria:

• child under 16 years old at enrolment into the study,

• newly diagnosed (within 18 months prior to the enrolment date) with an inflammatory rheumatic disease, including JIA, CTD, and vasculitis,

• registered as an outpatient with the pediatric rheumatology clinic,

• participation of a parent (mother, father, or guardian) in order to avoid bias (the parent will provide the satisfaction score on behalf of the child throughout the entire study period).

Exclusion criteria:

• children and parents who do not understand and speak French,

• no access to a telephone.

All children who had attended the pediatric rheumatology outpatient clinic from January 2010 to August 2012 were screened for eligibility in the study.

### Intervention

**Figure 1:**
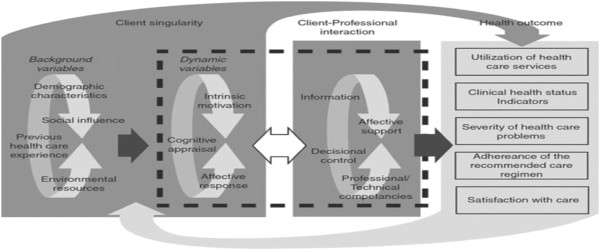
**The Cox’s Interaction Model of Client Health Behavior (IMCHB) reproduced with permission from [**[68]**].**

*The telenursing intervention* is based on Cox’s Interaction Model of Client Health Behavior (IMCHB) [67]. This model offers support in determining the optimal way for a nurse to interrelate with a patient to reach positive health outcomes. Its three conceptual foundations are: client singularity (individuals’ characteristics), client-professional interaction, and health outcomes [67,68] see Figure 1.

The TN intervention is designed to ensure continuity of care for children and their families through a telephone service providing nursing advice to meet families’ needs for health information, affective support, and help in decision-making [68]. The assumption underlying this parent-nurse interaction is that patient satisfaction is more likely when it is tailored to the unique needs of the patient/family client. The specific TN intervention is designed to ensure continuity of care for children and their families. This will be done via a telephone service providing nursing advice to meet families’ needs for: a) affective support; b) health information; and c) assistance with decision-making [68].

a. *Affective support*. This will involve the TN nurse making a follow-up call each month. The nurse will give the parent or adolescent time to speak and will listen attentively to their concerns. TN will also involve the nurse’s ability to calm any fears and meet the participant’s needs (parent/guardian or older children). The nurse’s ability to recognize the participant’s concerns is a predictor of satisfaction [69].

b. *Health information*. The TN nurse will provide information about the child’s health condition and explain treatments, medication, tests and the overall situation; the clarity of the information given is an important factor in satisfaction [69]. Information that is of great interest to parents (besides medical information) will be provided, including psychological impact, rehabilitation facilities, and alternative and complementary treatments [29].

c. *Aid to decision-making*. This important part of patient satisfaction is central to TN [68,70]. The TN nurse will facilitate parents’ involvement in making decisions by informing them of how their child’s care is progressing and presenting them with the different options that are likely to suit their needs and address their concerns [69,71,72].

Clinical knowledge, knowledge about the patient, experience, skills, understanding a multidisciplinary healthcare delivery system, and access to health information and resources, are all critical elements for the success of the telephone intervention and clients’ satisfaction [43,69,73]. Two specialist nurses, each with more than 5 years experience in adult and pediatric rheumatology, will attend a three-day course to enhance their skills in verbal communication, strategies for questioning parents and adolescents, assessing the quality of interactions, and aiding decision-making. The telephone intervention process will be standardized and recorded for each participant. It will involve a comprehensive, systemic assessment of the participant’s needs, including basic elements such as the participant’s characteristics, the date, time, and nature of the call, as well as a detailed description of the symptoms, problems, or reasons for the call. Following this assessment, prioritization and a plan will be developed collaboratively. Finally, the plan’s outcomes will be evaluated (was advice followed?).

Participants in the experimental TN group will attend a face-to-face medical and nursing consultation at baseline (T0) and then receive a monthly telephone call for 12 months. The face-to-face visit involves the TN nurse meeting with the parents and familiarizing herself with the child’s clinical, social, and family situation. The TN nurse will call the participant a total of 12 times, once during the last week of each month. Furthermore, the parent (and/or child if mature enough) will be given a telephone number (except for the control group) to call, as and when necessary, during normal Monday to Friday office hours. The TN nurse on duty will answer these calls and provide the same service.

#### Control group

Children in the control group will receive the same standard care and services provided to all children and their families admitted to the rheumatology outpatient clinic. Children with rheumatic diseases attend several appointments here and are followed up at varying time intervals depending on the progress of their disease, but usually four times a year. Currently, the medical management of these children is provided mainly by a pediatric rheumatologist, but other specialists participate as determined by the child’s needs. Physiotherapists and occupational therapists are available and can refer patients to healthcare professionals outside the hospital.

At T0, control group participants will also have a face-to-face medical consultation at the pediatric rheumatology outpatient clinic, during which the study protocol will be clarified. The medical consultation will be repeated every three months as per current practice. Parents will be informed that they can call the outpatient clinic when necessary and speak a duty nurse. To avoid potential contamination between groups, control group parents will be assigned a telephone number other than the TN service number. Currently, duty nurses are clinical specialists in pediatrics with some rheumatology experience, but are not specifically trained in TN. The nature and frequency of all calls will be recorded in a logbook.

### Data collection

**Figure 2:**
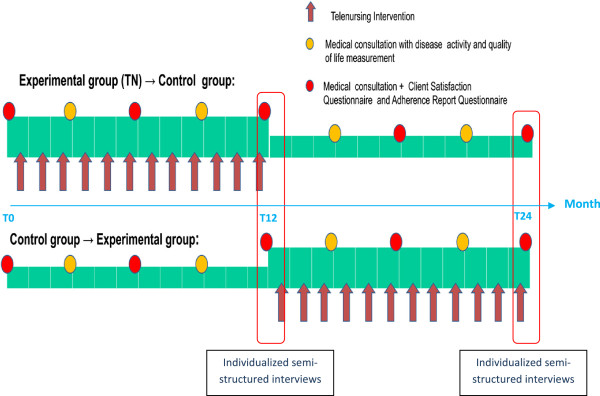
Telenursing intervention and data collection.

Data will be collected at different time points over the 24-month study period (see Figure 2). Participants switch to the other allocated group immediately after completion of study period 1. In this particular study, a wash-out period is considered unnecessary as the next measurement of satisfaction takes place six months after the start of period 2; this provides sufficient time to eliminate any residual effects of the treatment allocated in period 1. Data collection will be shared between the telephone nurses, a research assistant (nurse), and a physician. Telephone nurses will record the frequency, duration, and nature of all calls made and received. Records of each telephone call will be taken throughout data collection periods 1 and 2 (two years for each participant).The choice of the number of data collection points and the study timespan is based on theoretical and practical considerations [74]. For practical reasons, patients will be enrolled in the first week of the month; face-to-face consultation baseline data (demographics, health status, and satisfaction) will be collected at this time (T0). Collection points occur every three months for assessments of disease activity and health status (T3, T6, T9, T12, T15, T18, T21, and T24) and every six months for the satisfaction questionnaire (T6, T12, T18, and T24).

The qualitative data concerning satisfaction are collected through interviews at the end of period 1, at T12, and at the end of period, 2 at T24. Interviews will be conducted by an experienced research nurse in a non-judgmental and respectful manner, and at a predetermined location where privacy can be ensured. An interview guide with specific, prompting questions has been developed to guide the interviews. These questions are in relation to how parents felt supported through the service they received. Each interview will be tape recorded so as to accurately capture the participants’ comments and will transcribed verbatim using standardized transcription.

#### Study protocol compliance monitoring

The importance of compliance with the study will be emphasized when participants are recruited and periodically during the intervention period [75]. Parental and child compliance with their monthly telephone contacts and their scheduled visits to the outpatient clinic will be carefully monitored throughout the course of the study. Intervention group participants will be considered to have complied with the study protocol if at least 80% of scheduled TN calls have occurred. If non-compliance is noted, information will be gathered on underlying problems; the TN nurse will meet with a member of the research team and measures will be undertaken to rectify the situation. The same procedure will be used with participants who repeatedly fail to attend their appointments.

Newly diagnosed children may require more frequent medical consultations to monitor therapeutic and side effects. All consultations between scheduled study visits, as well as changes in treatment, will be recorded.

### Primary outcome measure

The study’s primary outcome is participant satisfaction (parent or adolescent). Cox’s IMCHB defines the concept of satisfaction with nursing care from the patient’s perspective. The degree to which nurses adapt their care to meet the specific needs of the parent, guardian, or child relates directly to satisfaction [68]. Satisfaction is assessed using a mixed methods approach where both quantitative and qualitative data are gathered and analyzed separately, and then the different results are converged.

Client Satisfaction Questionnaire-8 (CSQ-8) is an 8-item version of the 18-item CSQ developed by Attkisson and Zwick [76]. It was designed to measure global client satisfaction in service delivery and program evaluation; it is brief to administer, has good psychometric properties (as demonstrated in several studies), and has been translated and validated in several languages, including French [77]. CSQ-8’s items are rated on a 4-point Likert-type scale giving a total score ranging between 8 (no satisfaction) and 32 (total satisfaction). This study’s primary quantitative comparison will be the proportion of subjects in the intervention group (TN) and the control group (standard practice) with total CSQ-8 scores ≥ 30, as well as comparison within subjects as measured by CSQ-8 at T0, T6, T12, T18, and T24.

Qualitative data will be collected via individualized semi-structured interviews (SSI) at the end of each study period (T12 and T24) to avoid any bias. SSI have been commonly used in evaluating health programs and are particularly useful for exploring people’s in-depth knowledge, experiences and understanding. They can supplement or substantiate other sources of data [78]. Here, the objective of SSI will be to determine satisfaction with regards to affective support, health information, and support in decision-making received.

### Secondary outcomes measures

Measurement of the *Clinical health status* will be performed every three months as per standard practice. This will consist of a core set of standardized clinical assessments of disease activity and disease-specific quality-of-life measures. *Disease activity* will be measured using a core set of four measures using the standard, self-administered, disease-specific Juvenile Arthritis Disease Activity Score (JADAS). This includes: 1) a physician’s overall assessment of disease activity; 2) a patient/parent overall assessment of well-being (both measured using a 10 cm visual analogue scale); 3) the number of joints with active disease, and; 4) the erythrocyte sedimentation rate [79]. The equivalent available disease-specific core set of measures will be used for conditions other than JIA [31].

*Quality of life* will be measured using the French version of the Juvenile Arthritis Multidimensional Assessment Report (JAMAR) for parents (JAMAR-P) and for children aged between 11 and 18 years old (JAMAR-C). Both original versions of the JAMAR have been validated [80,81] and translated using standardized translation methods [82]. The JAMAR includes 15 patient-related outcomes: 1) a 15-item functional status questionnaire; 2) pain intensity; 3) a 10-item disease-specific quality-of-life outcome, including the physical and psychological domains; 4) child’s overall wellbeing; 5) presence of pain in joints; 6) morning stiffness; 7) presence of extra-articular symptoms (fever and rash); 8) perception of disease activity; 9) disease status at the time of visit; 10) evolution of the disease since previous visit; 11) list of medications taken; 12) medication side effects; 13) difficulties with medication administration; 14) school problems; 15) satisfaction with the illness outcome [83].

*Adherence to treatment* will be measured using the Parent Adherence Report Questionnaire (PARQ) and the Child Adherence Report Questionnaire (CARQ) that have both been validated in English and French [84,85]. The PARQ has been adapted to the local context with permission of the developers and includes four questions related to medication and exercise. Questions address perceived difficulties in following the various forms of treatment as well as the benefits of treatment. Each question is rated on a 10 cm visual analogue scale. The CARQ was developed from the PARQ to allow children ≥ 9 years old to respond to the questions themselves [85].

*Telenursing service utilization* will be recorded in terms of number, time, and duration of calls, who made them, the nature of the call, decisions taken, descriptions of the plan of action.

*Other outcomes*. Demographic data about participants in TN and other calls will be recorded, including age, gender, cultural background, marital status, occupation, education, language spoken at home, and types of treatment.

### Sample size and power

In 2008, 113 newly diagnosed children were admitted to the study hospital’s pediatric rheumatology outpatient clinic. Of these, 48 were diagnosed with an inflammatory rheumatic diseases. Based on these numbers, it is anticipated that around 70 children will be admitted to the clinic in the 18-month screening period prior to enrolment into the study. If we consider that 80% of patients/parents will give consent to their participation, this would leave a pool of 56 families available for the two-year study period.

A power analysis was calculated based on the number of participants expected to complete the study, not the number recruited initially. If we consider a difference in the proportion of subjects in the two groups with a satisfaction score ≥30, e.g. 70% in TN versus 20% in the control group, then 23 subjects per group (total of 46 subjects) would be required to reach a power level of .90 for an alpha level of .05 (two-sided test). Effective strategies will be used to maintain the sample size. To compensate for an expected attrition rate of 20%, two groups of 28 subjects will be recruited (total of 56 subjects).

### Randomization and allocation of treatment

In crossover trials, both experimental and control experiments are given to every participant, randomizing the order in which they are applied [65]. Consenting participants will be allocated treatment using a computer-generated simple block randomization. The benefits of using blocks are to ensure that the accrual of participants to either arm is uniform over time. An independent researcher, not involved in the recruitment or evaluation of follow-up, will prepare sealed, numbered envelopes containing an allocated treatment. Once randomization has occurred, blinding of participants and the caregiver is not feasible for this type of intervention, providing a potential risk for bias.

### Data analysis

The equivalence of the two groups will be checked using demographic variables such as age, marital status, occupation, level of education, cultural background, language, and child’s diagnosis. Differences between groups will be tested using chi-square and t-tests.

#### Hypotheses testing

We will carry out an ‘intent to treat’ analysis. That is, participants will remain in the group to which they were allocated or assigned, whether or not they are poor compliers with the TN intervention. Data will be summarized with the mean and SD; continuous variables will be tested for normality.

Data will be analyzed using mixed effect linear models to exclude any ‘carry-over’ and ‘temporal’ effect of the treatment and to test the time effect of TN. Data analyses will be performed a statistician using Stata version 13 software.

### Statistical methods

#### Mixed methods analyses

The primary outcome of satisfaction, as measured using the CSQ and the interviews, will be analyzed using the triangulation design convergence model described by Creswell [86]. This model is used for comparing results and corroborate quantitative results with qualitative findings. The quantitative and qualitative data will be analyzed separately, but the different results will be converged (by comparing and contrasting the results) for the interpretation of the findings. This method will provide valid and substantiated conclusions about the satisfaction outcome. Qualitative data analyses will be carried out using the content analysis method, including, a) identification of units of analysis, and b) analysis of content by developing categories using ATLAS.ti V6 computer software [87].

#### Data screening and missing data

Prior to data analyses, data will be screened for the accuracy of the data file, missing data, outliers, and distribution. Cases with more than 20% of missing data will be excluded from analyses and missing data will be randomly replaced using the expectation maximization (EM) method. “EM forms a missing data correlation matrix by assuming the shape of a distribution…for the partially missing data and basing inferences about missing values on the likelihood under that distribution” (p. 63) [88]. Normally distributed data and homogeneity of variance will be analyzed using parametric tests, and skewed data will be analyzed with the equivalent non-parametric tests.

#### Representativeness and bias

Demographic data and reasons for refusals to participate in the study, as well as those who are lost (withdraw, lost to follow-up, etc.…), will be compared to participants who remain in the study. Recruitment will be performed at the tertiary referral hospital for children with chronic rheumatic diseases in the Canton of Vaud, which will ensure the representativeness of the population of children with this condition and avoid referral bias. Furthermore, strategies to maximize the follow-up of study participants from both groups will be used. Finally, to minimize the risk for bias in self-reported satisfaction, participants will return their completed questionnaires in sealed envelopes addressed to the research team. Participants will also be informed that their satisfaction levels will be confidential, will not be shared with their caregivers, and will not interfere with the quantity and quality of care provided.

#### Natural maturation

will take place as each participant (adolescent and/or parent) becomes more familiar with the disease and treatment, which can potentially result in better satisfaction scores and outcomes. This aspect is dealt with in two ways. First, the crossover design with a randomized assignment of participants should decrease the risk of bias in the selection groups and result in similar distributions in each group. The adequacy of the follow-up durations for this study was determined in previous studies [14,74].

#### Contamination

Is not expected as each TN consultation is done on an individual basis. However, if contamination where to occur, this would result in better outcomes in the control group and would consequently reveal an apparent null effect of the intervention. We do not expect the usual high standards of care and services to change as a result of the introduction of TN as the nurse who gives the TN consultation is self-employed and provides the service on a private individual basis. Furthermore, a mobile telephone number will be used for the TN service, different from the outpatient clinic’s number assigned to the control group. To further control risk of contamination, only newly diagnosed children will be recruited into the study; they will thus not have had the opportunity to develop any relationships with the staff currently working at the study site. Nevertheless, all changes in policies and documentation will be recorded during the study period.

### Data management

#### Data quality

To ensure data quality, the research assistant will verify that all research forms have been completed at each data collection point of each data collection period. However, it is ultimately the Principal Investigator’s responsibility to ensure that appropriate work books and data sheets contain accurate and complete information.

#### Confidentiality and data security

Access to medical records will be limited to the research team and only with the approval of the Medical Director of the outpatient clinic. Measures to ensure data security and participant confidentiality will include: the removal of all identifying data and coding on the research forms; the storage of coded data on a password-protected computer with personal logins; access to data being limited exclusively to the research team; and data stored on paper or in recorded forms will be securely stored in a locked cabinet in the Principal Investigator’s office for a duration of 10 years after completion of the study [89].

### Ethical considerations

The study and its amendment were approved by the Human Research Ethics Committee on January 17, 2011 and March 28, 2011, respectively.

Families who met the inclusion criteria received an information letter by post about the research study and were informed that it will be discussed at their next appointment with the medical specialist. They received verbal information about the study’s objectives, its procedures, potential risks, and their right not to participate or to withdraw at any time without this affecting the quality of future care. They were also informed of the measures taken by the researcher to ensure confidentiality, including that the participants will not be individually identified by name in reports or publications. Upon agreement to participation in the study, parents will be asked to provide written informed consent. Written assent will be obtained from children who are considered to have the capacity to make an ‘informed decision’ about the intervention (from 11 to 16 years).

## Discussion

The literature review highlighted the paucity of studies demonstrating strong evidence of the benefits of TN consultations for children with chronic conditions. However, nurse-led telephone follow-up interventions have shown positive effects on treatment adherence in adult populations with chronic illness [90]. Treatment adherence to control children’s rheumatic diseases is crucial to limiting disability later in life. However, we know from the literature that when children enter adolescence, treatment adherence is poor, which may result in detrimental consequences [91]. Support to parents and adolescents is crucial in this difficult transition phase. Our intervention was designed to provide information, affective support, and aid to decision-making. Although this intervention has been standardized, it allows for individualized care because it is tailored to the client’s characteristics and needs ensuring regular follow-up care. The intervention is very much dependent on the competence of the person providing the consultation. It is clear that this type of interaction requires advanced specialized skills, which is the case with the nurses involved in our TN study.

This study’s design has several strengths. First, the combination of quantitative and qualitative data for the primary outcome (satisfaction) will provide a better understanding of how and why the participants are satisfied with the service and will provide additional validity to the results. Second, the study is conducted in several centers, representing a large proportion of the population of interest in western Switzerland. Third, the processes put in place for the quality control of study protocol compliance should be highlighted. All telephone calls are recorded and a checklist ensures that all elements of the intervention are provided. Close study monitoring also provides important information about the feasibility of the study intervention in real life situations. Finally, the research is an attempt to meet the needs of the population of children and adolescents with rheumatic diseases in western Switzerland. This topic is of foremost importance because, health outcomes have become a priority for hospital-wide quality improvement initiatives and the Swiss Nursing Research Agenda.

Some limitations must be acknowledged, however. The intervention made by two experienced nurses may be difficult to replicate by others if the necessary training and formal education is not provided to nurses. To the best of our knowledge, this type of education is not offered in current postgraduate nursing programs. Finally, the JAMAR questionnaires have been validated in their original versions only. Psychometric equivalence has yet to be determined and the results of this study will contribute to the further psychometric testing of this questionnaire. However, the rigorous translation method and pre-test should have minimized the risk of a change in the psychometric properties of the questionnaires.

### Trial status

Recruitment for the trial started in August 2011 and follow-up data collection is due to complete in August 2014.

## Abbreviations

TN: Telenursing; PROC: Pediatric rheumatology outpatient clinic; JIA: Juvenile idiopathic arthritis; CTD: Connective tissue diseases; IMCHB: Interaction model of client health behavior; CSQ-8: Client satisfaction questionnaire-8; SSI: Semi-structured interviews; JADAS: Juvenile arthritis diseases activity score; JAMAR: Juvenile arthritis multidimensional assessment report; PARQ: Parent adherence report questionnaire; CARQ: Child adherence report questionnaire.

## Competing interests

The authors declare that they have no competing interest.

## Authors’ contribution

ASR Study coordination, BF, JR, MH, CG Study design, ASR Manuscript drafting, BF, JR, CG, MH Manuscript review, ASR, BF, JR, CG ;MH Approval of the final manuscript

## Authors’ information

The Principal Investigator (ASR) is an associate professor at Institute of Higher Education and Nursing Research (Institut de Formation et Recherche en Soins-IUFRS) at the Faculty of Medicine and Biology, University of Lausanne, Switzerland. She also has a 0.2 FTE appointment as a Professor at the University of Applied Sciences and Arts Western Switzerland. She has more than 15 years of pediatric nursing experience and a strong background in clinical research and quantitative methods in particular. She teaches research methods to nursing master’s students and directs doctoral students at the IUFRS. She holds a PhD in nursing sciences from Curtin University of Technology, Western Australia. She has published in specialised scientific peer-reviewed journals. Her research in the nursing field has attracted funding from diverse sources.

The Co-Investigator (MH) is Privat-Docent and MER at the Medical Faculty of the University of Lausanne and head of the pediatric immuno-allergology and rheumatology unit of the CHUV in Lausanne. He is a consultant for rheumatology at the pediatric departments of the University Hospitals of Lausanne and Geneva, and Head of the Pediatric Rheumatology Network of Western Switzerland, providing consultations in Lausanne, Geneva, Sion, Neuchâtel and Aigle. He has 14 years of experience in pediatric rheumatology, he has established and developed pediatric rheumatology in the French-speaking part of Switzerland, and is considered an expert in this field nationally and internationally. In collaboration with the Ligue Genevoise contre le Rhumatisme, he developed a multidisciplinary approach for the care of pediatric patients suffering from rheumatism. He is a member of the council and treasurer of the Pediatric Rheumatology European Society (PReS), and he was vice-president (2003–2006) and president (2006–2009) of the Swiss Society of Pediatrics. He has teaching responsabilities at the University of Lausanne, at the HES in Geneva and the University of Lyon (DIU in pediatric rheumatology). With other colleagues, he has written the Swiss training program for the certification in pediatric rheumatology (“Schwerpunkt”), accepted by the FMH in 2008, and he leads the training centre for pediatric rheumatology of Lausanne-Geneva. Dr Hofer is actively involved in research and has been co-investigator in multi-centred studies (e.g. PRINTO and PFAPA syndrome studies). His research has also attracted competitive funding. He has published in specialized scientific peer-reviewed journals and is regularly invited to peer review manuscripts submitted for publication in the following journals: Arthritis and Rheumatism, Rheumatology, Journal of Rheumatology, Joint Bone spine, Clinical and Experimental Rheumatology, Clinical Rheumatology, Journal of Pediatrics, European Journal of Pediatrics and Acta Paediatrica. Recently, he received a Research Award from the SOFREMIP (Sté Francophone Rhumatologie Pédiatrique) for the project entitled: “International registry for PFAPA syndrome: prospective evaluation of a cohort of patients”.

The Co-Investigator (BF) has been the Director of the Geneva League for Rheumatology, Switzerland (Ligue Genevoise contre le Rhumatisme), since 2001. She is a registered nurse with a solid experience in caring for children with rheumatic diseases and supporting their families. In her leadership role at the League, she has greatly contributed to the development of a specialist level nursing role in pediatric and adult rheumatology. Her role involves supporting children and their families not only in hospital settings, but also in the community. Her strong clinical skills and knowledge of these children and their families, as well as being a specialist in providing telenursing support, has been critical to the conduct of this study.

The Co-Investigator (JR) is head nurse of the pediatric outpatient service in the University Hospital Centre of Lausanne (CHUV), Switzerland. He has more than 14 years of pediatric nursing experience and has a strong background in clinical practice. He holds an MSc in Nursing Sciences (administration), from Montreal University, Canada. He has participated in different clinical research studies.

The Co-author (CG) is a master-prepared registered nurse with more than 11 years of intensive care experience. Since 2010, he has worked at the University of Applied Sciences and Arts Western Switzerland (HESAV), first as research assistant for this study, and now also as lecturer in the bachelor’s programme.

## Pre-publication history

The pre-publication history for this paper can be accessed here:

http://www.biomedcentral.com/1471-2431/14/151/prepub
